# Natural and hybrid immunity after SARS-CoV-2 infection in children and adolescents

**DOI:** 10.1007/s15010-024-02225-w

**Published:** 2024-03-18

**Authors:** T. Rothoeft, C. Maier, A. Talarico, A. Hoffmann, A. Schlegtendal, B. Lange, A. Petersmann, R. Denz, N. Timmesfeld, N. Toepfner, E. Vidal-Blanco, S. Pfaender, T. Lücke, F. Brinkmann

**Affiliations:** 1 University Hospital of Pediatrics and Adolescent Medicine, St. Josef-Hospital, Ruhr-University, Bochum, Germany; 2 Department of Epidemiology, Helmholtz Centre for Infection Research, Brunswick, Germany; 3 University Institute of Clinical Chemistry and Laboratory Medicine, University Medicine Oldenburg, Oldenburg, Germany; 4 Department of Medical Informatics, Biometry and Epidemiology, Ruhr-University Bochum, Bochum, Germany; 5 Department of Pediatrics, Faculty of Medicine, University Hospital Carl Gustav Carus, Technische Universität Dresden, Dresden, Germany; 6 Department of Molecular and Medical Virology, Ruhr University Bochum, Bochum, Germany; 7 University Children’s Hospital, Lübeck, Germany; 8 University Institute of Clinical Chemistry and Laboratory Medicine, University Medicine Greifswald, Greifswald, Germany; 9 Airway Research Center North (ARCN), German Center for Lung Research (DZL), Lübeck, Germany

**Keywords:** SARS-CoV-2, Immunity, COVID-19, Children, T cell, Antibody, Convalescent, Vaccination

## Abstract

**Purpose:**

In contrast to adults, immune protection against SARS-CoV-2 in children and adolescents with natural or hybrid immunity is still poorly understood. The aim of this study was to analyze different immune compartments in different age groups and whether humoral immune reactions correlate with a cellular immune response.

**Methods:**

72 children and adolescents with a preceding SARS-CoV-2 infection were recruited. 37 were vaccinated with an RNA vaccine (BNT162b2). Humoral immunity was analyzed 3–26 months (median 10 months) after infection by measuring Spike protein (S), nucleocapsid (NCP), and neutralizing antibodies (nAB). Cellular immunity was analyzed using a SARS-CoV-2-specific interferon-γ release assay (IGRA).

**Results:**

All children and adolescents had S antibodies; titers were higher in those with hybrid immunity (14,900 BAU/ml vs. 2118 BAU/ml). NCP antibodies were detectable in > 90%. Neutralizing antibodies (nAB) were more frequently detected (90%) with higher titers (1914 RLU) in adolescents with hybrid immunity than in children with natural immunity (62.5%, 476 RLU). Children with natural immunity were less likely to have reactive IGRAs (43.8%) than adolescents with hybrid immunity (85%). The amount of interferon-γ released by T cells was comparable in natural and hybrid immunity.

**Conclusion:**

Spike antibodies are the most reliable markers to monitor an immune reaction against SARS-CoV-2. High antibody titers of spike antibodies and nAB correlated with cellular immunity, a phenomenon found only in adolescents with hybrid immunity. Hybrid immunity is associated with markedly higher antibody titers and a higher probability of a cellular immune response than a natural immunity.

## Introduction

Children infected with SARS-CoV-2 (severe acute respiratory syndrome coronavirus 2) are mostly asymptomatic or develop much less severe coronavirus disease 2019 (COVID-19) than adults [1]. Immune responses to COVID-19 in adults and children probably differ, as children have a higher steady-state expression of IFN-γ response genes [2, 3], especially in their upper respiratory tract. This may reduce virus replication and lead to faster clearance in children. The systemic immune response in blood is characterized by a more naïve state [4] compared to adults. The extend of NCP antibody titers is highly variable following SARS-CoV-2 infection in children; according to data from our current follow-up study (Corkid 2.0) [5] up to 27% of cases have no or very low NCP antibody titers < 10 IU/ml, albeit PCR-confirmed SARS-CoV-2 infections. Studies with detection of SARS-Cov-2-specific T cells by IGRA in adults after infections or vaccination show that virus-specific T cells can be detected even after asymptomatic or mild infections, even if no seroconversion has been induced [6, 7]. Also, these virus-specific T cells appear to persist longer than virus-specific antibodies [8, 9].

In this study, children and adolescents with a SARS-CoV-2 infection confirmed by PCR or rapid antigen test and those vaccinated with an RNA vaccine (BNT162b2, Comirnaty®) with subsequent SARS-CoV-2 infection (hybrid immunity) were examined 3–26 months (median 10 months) after infection. The humoral immune response against SARS-CoV-2 (nucleocapsid-specific antibodies, spike protein-specific antibodies, and neutralization assays) and the cellular immune response (interferon-γ release assay) were characterized.

## Methods

### Participant characteristics

All participants had already taken part in the population-based Corkid study two years previously [10] and in a follow-up study with determination of SARS-CoV-2-specific antibodies from October to December 2022 as part of the Immunebridge Study of the German Network University Medicine [11, 12]. All 259 participants of the last study were invited by mail to participate in the current study with the specific question of cellular and humoral immunity following a SARS-CoV-2 infection. 128 were willing to participate, of which 71 could be tested for Spike and NCP antibodies from October to December 2022 and 76 could be studied in January and February 2023. 4 of the 76 children (mostly < 6 years) had to be excluded due to the lack of material for IGRA and nAB determination. 32 participants were children (aged 4–10 years), 40 were adolescents (aged 11–21 years). 37 participants were vaccinated against SARS-CoV-2, 86.5% of vaccinated the participants received two or more doses of BNT162b2 (Table 1).

**Table 1:** Number of vaccination doses administered in different age groups

Number of vaccination doses	Number of vaccinated participants		
	All	Age in years	
	37	4–10	11–21
1	5	2	3
2	19	5	14
3	13	0	13

For all participants, background information were known from the population-based Corkid database from 2020/21 [5, 10] and updated in 2023. Vaccinations, chronic illnesses, and the type of chronic medication as well as the onset of known SARS-CoV-2-infections, the type of detection (PCR; rapid antigen or AB), the severity of symptoms (classification: asymptomatic; mild in case of symptoms such as a normal cold; moderate in case of fever and cough lasting several days, shortness of breath, and similarly severe symptoms; severe in case of hospitalization) had been inquired from the treating physicians and parents by a standardized questionnaire. Demographic as well as the relevant clinical data of the 72 children are presented in Table 2.

**Table 2:** Clinical and demographic data of all participants, divided in those with positive or negative neutralizing antibodies (gray rows), and IGRA response (blue rows)

	All	Neutralizing antibodies			IGRA response		
		Positive	Negative	OR (95% CI)	Positive	Negative	OR (95% CI)
*N*	72	56	15		48	23	
Female (%)	34 (47.2%)	24	9		23	10	
Age in years	11 (7)	12 (7)	6 (4)	***p* < 0.001**	12 (5.25)	7 (4)	***p* < 0.001**
Children (4–10 years)	32	20 (62.5%)	12 (37.5%)	Ref.	14 (43.8%)	17 (53.1%)	Ref.
Adolescents (11–21 years)	40	36 (90%)	3 (7.5%)	**7.2 (1.8–28.6)**	34 (85%)	6 (15%)	**6.9 (2.2–21.1)**
Chronic disease	16	14 (87.5%)	2 (12.5%)	2.2	9 (56.3%)	7 (43.8%)	0.5 (0.2–1.7)
With medical treatment	8	8 (100%)	0	–	6 (75%)	2 (25%)	1.5 (0.3–8.1)
Severity of SARSCoV 2 infection							
Asymptomatic	14	12 (85.7%)	2 (14.3%)	1.8 (0.4–9)	9 (64.3%)	5 (35.7%)	0.8 (0.2–2.8)
Symptomatic	55	42 (76.4%)	12 (21.8%)	0.8 (0.2–3)	38 (69.1%)	16 (29.1%)	1.7 (0.5–5.1)
Vaccination	37	37 (100%)	0	–	32 (86.5%)	5 (13.5%)	**7.2 (2.3–22.9)**
Children (4–10 years)	7	7 (100%)	0	–	5 (71.4%)	2 (28.6%)	Ref
Adolescents (11–21 years)	30	30 (100%)	0	–	27 (90%)	3 (10%)	3.6 (0.5–27.4)
Time (months) from vaccination to 2nd assessment	12 (2)	12 (2)	–	–	12 (2)	12 (2)	n.s

72 children took part, in one patient neutralizing antibodies could not be determined to the lack of material and in another patient the IGRA could not be performed (age and times: median (interquartile range) percentages refer to 3rd line; bold: significant differences between positive/negative response, *ys* years, *CI* confidence interval)

### Antibody measurement

Antibody measurements were conducted using electrochemiluminescence immunoassay (Elecsys Anti-SARS-CoV-2, cobas pro, Roche Diagnostics GmbH, Mannheim, Germany). SARS antibody test against spike (S) and nucleocapsid (N) protein were based on IgG and IgM. SARS-CoV-2 spike (S) protein antibodies were assessed quantitatively. Values ≥ 0.8 binding antibody units (BAU)/ml) were considered positive for SARS-CoV-2 spike (S) protein antibodies. Measurements of nucleocapsid (NCP) protein antibodies were assessed qualitatively and considered positive if values were above the assay-specific cut-off index [COI] ≥ 1.0 IU/ml.

### Virus-neutralization assay

A propagation-defective vesicular stomatitis virus (VSV) pseudovirus-based neutralization assay was used to determine SARS-CoV-2 neutralizing antibodies as previously described [13]. To this end pseudotype viruses expressing the wild-type SARS-CoV-2 spike (S) protein (SΔ18 (codon-optimized, C-terminal truncation of 18 amino acid residues, GISAID Accession ID: YP_009724390.1) were used. To determine pseudotype virus neutralization (PVN), patient sera were first complement-inactivated at 56 °C for 30 min. Patient sera were serially diluted from 1:20 (lower limit of detection) to 1:2560 (upper limit of detection) in triplicates and incubated with pseudo virus for an hour at 37 °C. After incubation, the suspension was transferred to Vero E6 cells, that had been previously seeded at a density of 1 × 10^5^ cells per mL in a 96-well plate, and incubated overnight. Samples were lysed and the luciferase activity determined as relative light units (RLU). The antibody dilution resulting in a 50% decrease in luminescence, corresponding to 50% PVN (PVND 50), was calculated using GraphPad Prism (Version 9.5.1).

### QuantiFERON-SARS-CoV-2 ELISA

The SARS-CoV-2-specific interferon-γ releasing response was obtained using the QuantiFERON-SARS-CoV-2 assay [14]. 4 ml of whole blood was collected into Lithium-Heparin tubes and stored at 5–8 °C before being transferred to specialized QFT-Plus Blood Collection within 52 h. Aliquoted samples were incubated for 24 h at 37 °C (a Nil tube as negative control, a mitogen tube as positive control, and one peptide-containing tube with SARS CoV-2 proteins RBD, S1, S2, N, M, NSP) to stimulate the immune cells. After incubation, the tubes were centrifuged at 2500 × *g* for 15 min and the plasma separated. Then, levels of IFN-γ were measured for the plasma samples by a chemiluminescence immunoassay (Liaison XL, DiaSorin GmbH, Dietzenbach, Germany). The QFN-SARS results were interpreted according to the manufacturer’s specifications. IFN-γ values (in IU/ml) for Ag1, Ag2, and mitogen were corrected for background by subtracting the IU/ml value obtained for the respective Nil control and IGRA were scored as reactive if the corrected IFN-γ values were ≥ 0.15 IU/ml and ≥ 25% of Nil. Otherwise, they were scored as non-reactive. Values > 10 IU/ml are reported as > 10 IU/ml.

### Statistics

For the statistical analysis, the severity of the SARS Cov2 was categorized as “asymptomatic” or “symptomatic”. Age was divided into two groups for descriptive purposes and used as a continuous variable in multivariate analyses. The classification of the level of NCP antibodies into negative or low, medium, and high values was based on data from 177 children in the recent follow-up study (Immunebridge) from 2022 [11, 12].

This prospective study was planned to determine the relationship between NCP antibodies and the IGRA values. Because of this, all univariate analyses have exploratory character; *p*-values are, therefore, only for orientation and have no confirmatory significance. The frequency between children with or without IGRA response, the odds ratio, and 95% confidence interval were calculated.

Using the R programming language (version 4.2), immune responses were analyzed both quantitatively and qualitatively using crude and adjusted logistic and linear regression models, respectively. Both types of regression models were calculated with and without adjustment for age, vaccination status, and time since last covid-19 infection.

## Results

### SARS-Cov2 history

All 72 children and adolescents had at least one infection during February–June 2022 in the Omicron era in Germany detected by rapid antigen test (26%) or PCR (74%). Only one female adolescent had already suffered from a SARS-CoV-2 infection in 2020, but she reported a reinfection in 2022. The interval between this study and the last infection was 3–26 months (median 10 months).

In 20% of individuals, the infection was asymptomatic, in most cases mild (70%) or at most moderate (10%), no participant was hospitalized. From January to April 2022, the participants were vaccinated with the mRNA vaccine BNT162b2, the only approved vaccine for children in Germany at this time. 37 (53%) were vaccinated, 7 (22%) of the group of children (4–10 years), and 30 (75%) of the group of adolescents (11–21 years) (Table 2).

### Antibody titers

#### Nucleocapsid antibodies

**Fig. 1:**
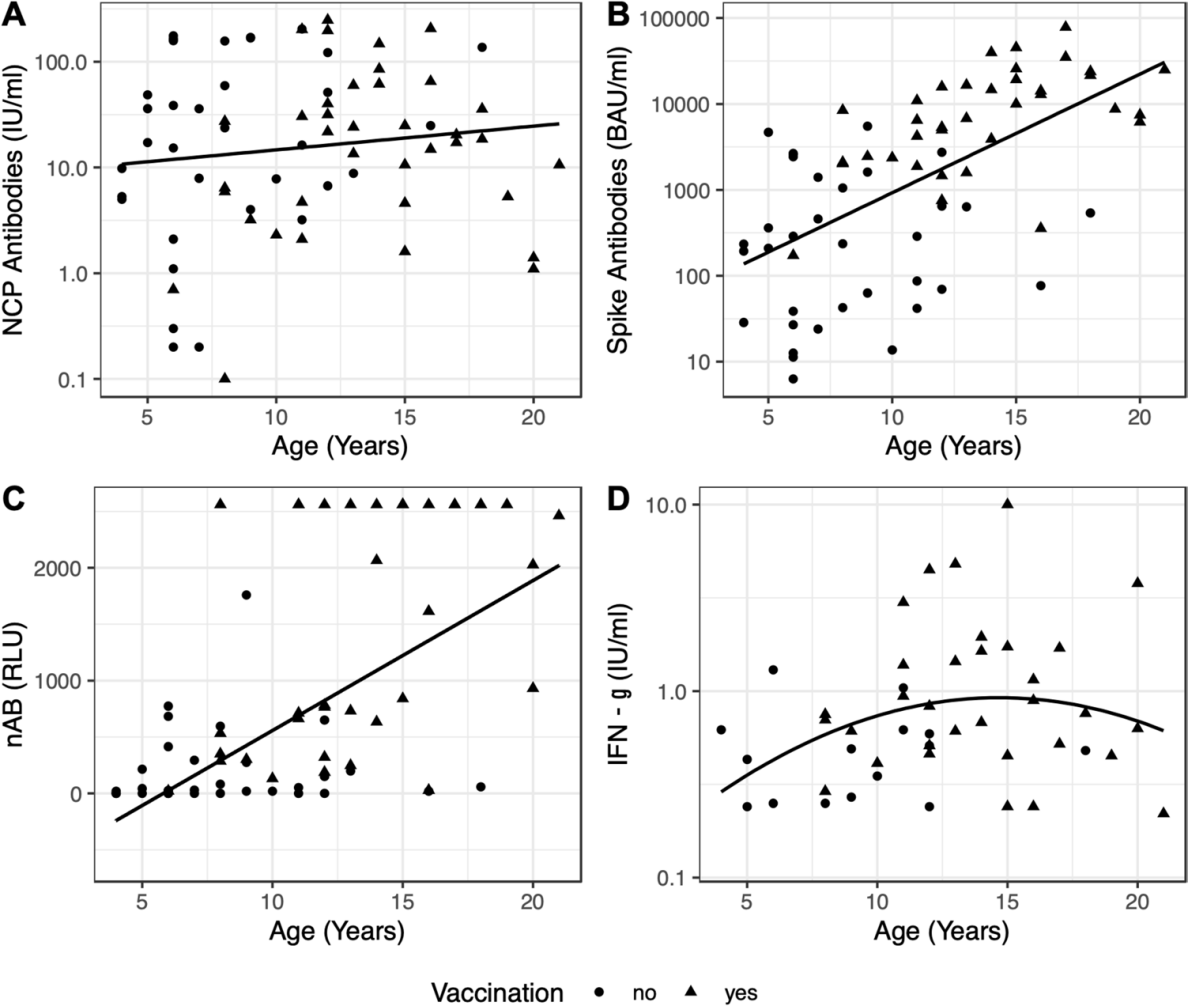
The NCP antibody titers were independent of age and vaccination (**a**). Spike protein-specific antibodies were detectable in all participating children. The titer was 50-fold higher in the vaccinated participants (**b**). nAB levels were significantly higher in vaccinated adolescents than in children < 10 years of age (**c**). Age and vaccination status had no effect on the amount of interferon-γ released by T cells (**d**). Logarithmic scales are used for **a**, **c** and **d**. **a**, **b** are simple linear regressions, **c** is a linear regression fitted using a rank-based estimation, **d** is a linear regression with a quadratic term. All regression lines are shown with 95% confidence intervals

Antibodies were analyzed in October–December 2022 and January–February 2023. In the 1st assessment, 4 children aged 5–10 years had no detectable NCP antibodies; in the 2nd assessment, NCP antibodies were not detectable in 5 children aged 5–10 years (Table 3) despite a PCR-confirmed mild or asymptomatic SARS-CoV-2 infection. All of these children had detectable spike protein-specific antibodies. Two of these children were also vaccinated (one received one dose, the other two doses). All children who had no NCP antibody in the 1st assessment were also negative in the 2nd assessment, one child had a very low NCP antibody titer in the first assessment. The decrease of NCP antibody titers within 3 months was on average very small (median from 33 to 23 IU/ml). In 21/24 children (30%/33%), the NCP antibody titer slightly increased over time. The NCP antibody titers in 2023 were independent of age, gender, vaccination, and time interval to the last known SARS infection (Fig. 1).

**Table 3:** Distribution of SARS nucleocapsid, spike protein, neutralizing antibodies, and IGRA in children and adolescents

	All	Nucleocapsid AB (IU/ml)		Spike protein AB (BAU/ml)		Neutralizing AB (RLU)		IGRA	
		Positive	Negative	Positive	Negative	Positive	Negative	Reactive	Non-reactive
1st Assessment (Oct/Dec 2022)	71	66 (93%)	5 (7%)	70 (98.6%)	1 (1.4%)	–	–	–	–
Titer Median (ICR)		32.2 (67.9)	–	1356.5 (10.245)	–	–	–	–	–
2nd Assessment (Jan/Feb 2023)	72	66 (91.7%)	6 (8.3%)	72 (100%)	0 (0%)	56 (78.9%)	15 (21.1%)	48 (67.6%)	23 (32.4%)
Titer median (ICR)		22.8 (54.7)	–	2061.5 (7948)	–	697.9 (2320)	–	0.6 (0.7)	–
Titer in different age groups median (ICR)									
Children (4–10 years)	32	17.2 (48)	–	325 (2118)	–	290.2 (476)	–	0.4 (0.3)	–
Adolescents (11–21 years)	40	24.1 (54)	–	6647.5 (14.900)	–	1820.5 (1914)	–	0.7 (1)	–

71 children took part in the first assessment, 72 children in the second assessment. In one patient, neutralizing antibodies could not be determined to the lack of material and in another patient, the IGRA could not be performed (titer: median (interquartile range); bold: number of positive and negative children and adolescents, *ys* years; *IGRA* Interferon-γ release assay)

#### Spike protein-specific antibodies

Spike protein (S)-specific antibodies were detected in all 72 children. The titer was 50-fold higher in the vaccinated participants (median 7488 vs 288 BAU/ml; *p* < 0.01, Fig. 1). Within 3-month interval between the two assessments, the S antibody titer slightly decreased in most children with a hybrid immunity; in the group of non-vaccinated children, a small but significant increase was observed (Table 3).

#### Neutralizing antibodies (nAB)

Neutralization assays were performed in January–February 2023. nAB were positive in 56 of the 71 (79%) assessed children. The detection of nAB was significantly more probable in children with a hybrid immunity compared to those with a natural immunity (Table 2), as all vaccinated children had neutralizing antibodies (nAB). nAB levels were significantly higher in vaccinated adolescents (Table 3) than in children (Fig. 1).

### IGRA

**Fig. 2:**
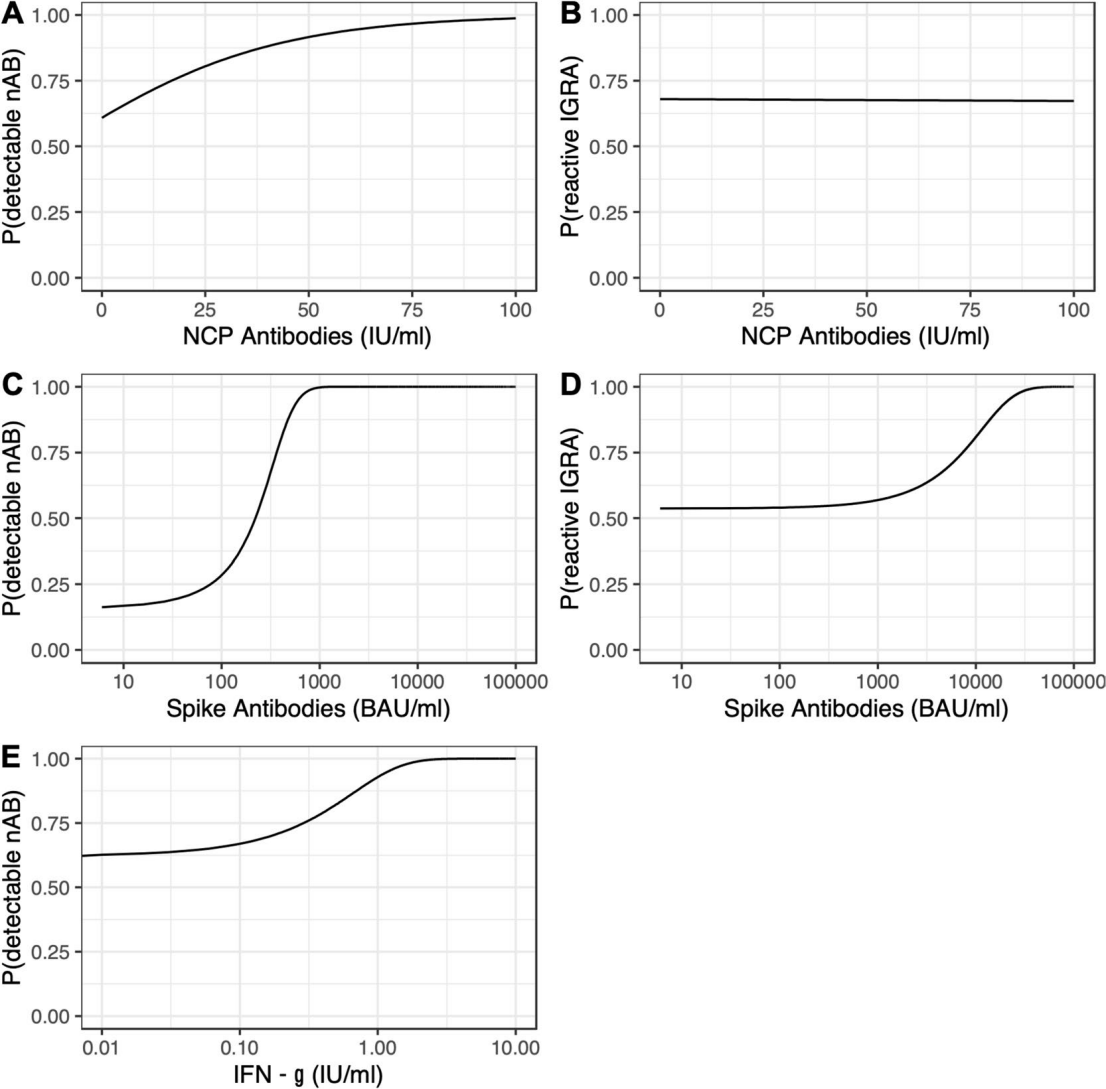
Detectable NCP antibodies were neither correlated with other antibodies nor with cellular immunity (**a**, **b**). High titers of Spike antibodies (> 1000 BAU/ml) were associated with a higher probability for the presence of neutralizing antibodies (**c**). Extremely high titers of Spike antibodies (> 10,000 BAU/ml) were also associated with a higher probability of positive interferon-γ release assays (**d**). The presence of nAB was also conjoined with an increased likelihood for interferon-γ release in T cells (**e**)

IGRAs were measured in January–February 2023. IGRAs were non-reactive in 23 participants (33%), among them were 4 children who had no detectable NCP antibodies, but 19 children with detectable NCP antibodies. The probability of a reactive IGRA increased fivefold in adolescents compared to younger children (Table 2). The intensity of preceding infections may also influence the induction of a cellular immune response. In children, 38% of the children with asymptomatic infections had non-reactive IGRA, whereas 48% of those with symptomatic infections had a reactive IGRA, although this is not statistically significant due to the small number of participants in this age group. Age and vaccination status had no effect on the amount of interferon-γ released by T cells (Fig. 2).

### Interdependency of cellular and humoral immunity

Interdependencies between the different components of immunological memory were analyzed. Detectable NCP antibodies were neither correlated with other antibodies nor with cellular immunity. The presence of S antibodies did not correlate with NCP antibodies, but high titers of S antibodies (> 1000 BAU/ml) were associated with a higher probability for the presence of neutralizing antibodies. Extremely high titers of S antibodies (> 10,000 BAU/ml) were also associated with a higher probability of positive interferon-γ release assays. The presence of nAB was also conjoined with an increased likelihood for interferon-γ release in T cells (Fig. 2).

## Discussion

This study investigated humoral and cellular immunity against SARS-CoV-2 in children and adolescents with natural or hybrid immunity.

In most children having experienced mild or even asymptomatic SARS-CoV-2 infections, NCP antibodies persist for nearly one year after infection, although titer height is highly variable. Several studies in adults show a decline in NCP antibody titers beginning 4–6 months after the onset of symptoms with insignificant titers ten months after infection [15–18], a finding in line with one other pediatric cohort [19]. A small reduction in median titer of NCP antibodies was observed over the course of three months in our cohort, confirming previous findings at least in part. In some children eight years or younger we could not detect NCP antibodies, a phenomenon we did not see in older children; otherwise, the NCP antibody titers did not correlate with the age of the participants. As expected, NCP antibody titers were not affected by vaccination. As we tested twice with an interval of more than twelve weeks a delayed humoral response cannot be attributed for the lack of NCP antibodies; the absence of seroconversion albeit proven infection is an already described phenomenon in less severe SARS-CoV-2 infections [20]. As we found this phenomenon only in younger children it is tempting to speculate on pre-existing antibodies against N proteins of other human coronaviruses in these children that may protect to some degree against SARS-CoV-2 infections and thus lead to less severe infections inhibiting antibody production [21].

S antibodies were detectable in all children and adolescents, with titers being 50-fold higher in vaccinated participants. There may also be correlation of titer height with age, but a larger sample size with more vaccinated and unvaccinated individuals of different ages would be necessary to disentangle these effects. S antibodies could also be detected in younger non-vaccinated children who were tested negative for NCP antibodies as well as for Interferon-γ production in T cells. In asymptomatic or mild wild-type infections in adults, seronegativity is described in 1–9% of patients [22–25]. We could not confirm these results, but this may be due to the relatively small sample size.

The increasing S and NCP antibody titers observed in some non-vaccinated children may be attributed to boosting by clinically inapparent SARS-CoV-2 reinfections.

All participants with a hybrid immunity had neutralizing antibodies in contrast to only 50% of those with a natural immunity following an infection in the Omicron era. Moreover, we found significantly increased neutralization capacities in adolescents with a hybrid immunity, which is not surprising as this pattern is already described in adults [26–28] and resembles a heterologous prime-boost-boost regimen [29]. Whether this higher neutralization capacity in adolescents is an effect of age or vaccination cannot be answered due to the small sample size and the different vaccination rates in the different age groups. In another small study, non-vaccinated convalescent children had lower mean nAB titers than non-vaccinated convalescent adults. Adults with hybrid immunity had much higher titers than adults with a natural immunity, making effects of both vaccination and age possible [30]. Ouyang and colleagues describe lower titers of neutralizing antibodies in convalescent children with natural immunity compared to adults with natural immunity after BBX-infections, supporting the notion this may be an age dependent effect [31].

We did not elicit from our data the sequence of infection and vaccination, but at least in adults this is not relevant for the resulting immune response [28, 32].

The QFN-Test was validated [33] in patients infected with SARS-CoV-2 variant B.1.1.7 (Alpha) and its usability was also shown in the Omicron era [30]. More than half of the non-vaccinated convalescent children had a non-reactive interferon-γ release assay, but some exhibited a robust cellular immune response. This finding is somewhat contradictory to the results of other studies, but data on this immune reaction following an Omicron infection are sparse. In one small study group, convalescent unvaccinated pediatric and adult participants were described as totally IGRA negative [30] 10 months after infection; whereas in another small cohort of unvaccinated adult following infection with Omicron, these were mostly positive for T cell-mediated immunity while being seronegative [6].

Vaccinated adolescents with a hybrid immunity following an Omicron infection had a higher probability of having a reactive IGRA. Again, if this effect is only caused by vaccination or is also dependent on the age of the probands cannot be deduced from this study. An at least partial vaccination effect is probable, as a significantly higher percentage of reactive IGRAs in adults with a hybrid immunity compared to those with a natural immunity is described [30, 34].

Then, again, an age dependency with a lower frequency of SARS-CoV-2-specific T cells in children than in adults was also seen in small pediatric study groups infected with the ancestral strain utilizing flow cytometry to detect antigen-specific T cells [19, 35, 36]. In contrast to our study, specific memory T cells were detectable in these cohorts one year after a mild or asymptomatic SARS-CoV-2 infection. The difference to our results is probably attributable to a different method used in these studies to trace memory T cells, as flow cytometry-based assays are shown to have a greater sensitivity in detecting SARS-CoV-2-specific T cell responses [37, 38] than IGRAs. Furthermore, IGRA levels are known to decrease over time [39]. The phenomenon of an age-dependent diminished sensitivity is also described in interferon-γ release assays for the detection of Mycobacterium tuberculosis in children [40, 41]. This raises the question whether younger children are less likely to have SARS-CoV-2 specific memory T cells following infection or whether the IGRA test has weaknesses in monitoring cellular immunity in younger children. As whole blood is used for the QuantiFERON interferon-γ release assay, containing other immune factors such as memory T cells, this phenomenon may be attributable to the fact that younger children have a higher percentage of naïve T cells in peripheral blood [36] therefore interfering with bystander activation of memory CD8^+^ T cells by cytokine stimulation [42], which is an important aspect of immune responses to pathogens [43]. If this constraint may be overcome by adapting cut-off values in children without impairing specificity needs further studies.

A small influence of the severity of prior SARS-CoV-2 infections cannot be ruled out, as 38% of the younger children with asymptomatic infection had a reactive IGRA versus 48% of the younger children after a symptomatic SARS-CoV-2 infection. But again, the study is not sufficiently powered to control so many variables simultaneously.

Ten months after acquiring natural or hybrid immunity, the different compartments of immune memory show different reactions with complex relationships between different aspects of immune memory. Nucleocapsid antibodies were, irrespective of titer, not qualitatively and quantitatively predictive of memory T cells [23] determined by interferon-γ release as described before. The presence of nAB correlates with cellular immunity, as shown in one previous study [44]. Detectable spike protein antibodies were conjoined with interferon-γ release by T cells when these titers were extremely high; titers of this magnitude were only found in adolescents with a hybrid immunity.

Our findings support the concept of vaccination of convalescent children to further enhance the immune response. Whether this boosted immunological memory results in fewer or less symptomatic SARS-CoV-2 infections with other VOCs is a question not answered by this study.

The presence of high titers of spike antibodies and nAB were associated with an increased likelihood for the detection of interferon-γ release by T cells. The time interval to the last known SARS infection did not affect the detection of spike antibodies, nAB and interferon-γ release by T cells.

## Conclusion

**Fig. 3:**
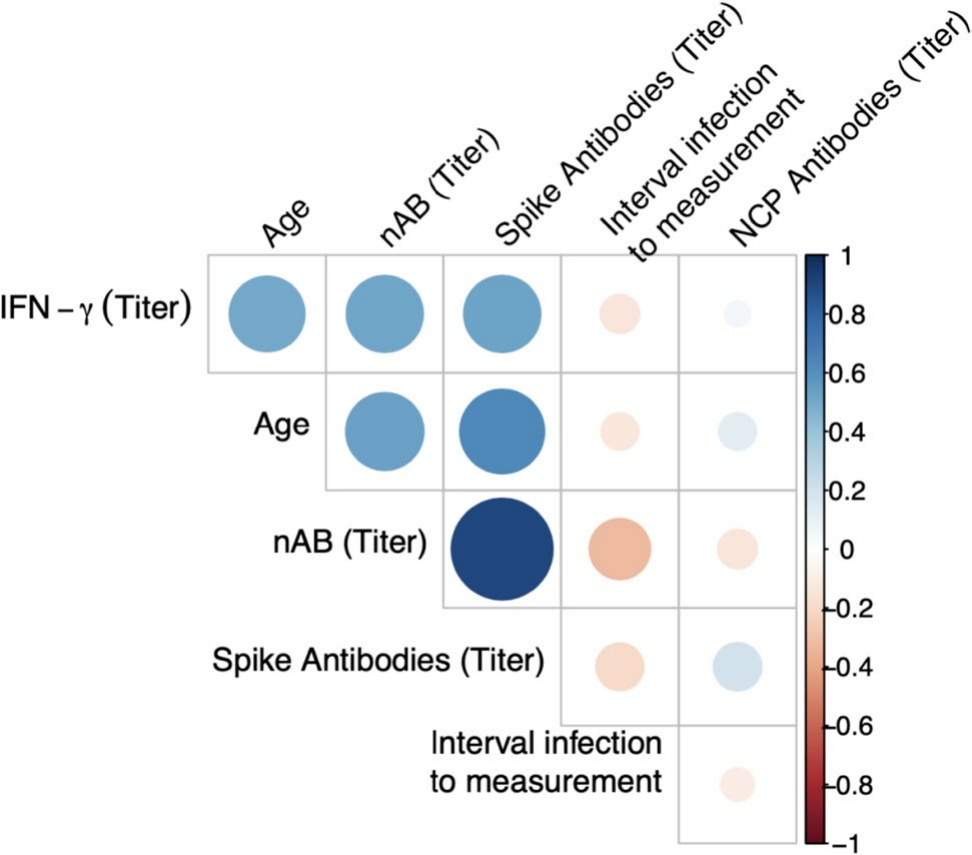
The NCP antibody titers were independent of age and time interval to the last known SARS-infection. Detection of NCP antibodies did neither correlate with the presence of other antibodies nor with cellular immunity. Spike antibodies and nAB had a higher probability of being detected in large amounts in adolescents with a hybrid immunity. In those participants with high spike antibody titers, nAb had a high probability of also being detected

S-protein and NUC-specific antibodies may be the most reliable marker to monitor immunity following SARS-CoV-2 infection and vaccination in children. A specific humoral immune response as measured by NCP antibodies was not predictive of a cellular immune response measurable by IGRA. In adolescents with a hybrid immunity, high antibody titers of spike antibodies and nAB were predictive for a cellular immune reaction. A hybrid immunity is associated with markedly higher antibody titers and a higher probability of a cellular immune response than a natural immunity (Fig. 3).

## Limitations

A major limitation of our study is the relatively small sample size of our cohort. Whether wild-type infections or infections by other VOC may cause differing immune reactions in children is a question not addressed by this study, as all but one infection took place in early 2022, when the Omicron variant was the predominant VOC. All analyses were performed with assays validated in wild-type infections. This may especially affect the neutralization assay, as Omicron infections are known to induce antibodies with a lower neutralization capacity against the wild-type virus [44]. We cannot rule out interfering clinically inapparent SARS-CoV-2 reinfection boosting antibody production or cellular immunity.
